# Health Monitoring of Laboratory Rodent Colonies—Talking about (R)evolution

**DOI:** 10.3390/ani11051410

**Published:** 2021-05-14

**Authors:** Stephanie Buchheister, André Bleich

**Affiliations:** Institute for Laboratory Animal Science, Hannover Medical School, Carl-Neuberg-Str. 1, 30625 Hannover, Germany; buchheister.stephanie@mh-hannover.de

**Keywords:** health monitoring, hygienic standardization, microbiological diagnostics, rodent pathogens, exhaust air dust PCR, research validity, microbiome analysis, metagenomic analysis

## Abstract

**Simple Summary:**

When working with laboratory animals, scientists need to ensure that animals are not suffering from natural infections. Therefore, hygienic standards have been developed over the last 100+ years. The key element of hygienic standardization is the monitoring of infectious agents that can compromise the animal’s health, are dangerous for the personnel or interfere with the research. However, scientists became aware that by eliminating such unwanted infectious agents the overall diversity of all microbes in research animals, the so-called microbiome, has also been reduced. Moreover, it became clear that the microbiome composition has an enormous impact on how research models react, e.g., to treatments. This might hinder the translation of findings in preclinical research to the clinical situation. Therefore, new concepts in hygienic standardization need to be developed that take animal welfare as well as scientific value into account. In this review, we will give an overview of how classical monitoring has been evolved and demonstrate concepts regarding how to handle the microbiome as a yet unknown variable in animal-based research in order to enhance the validity of research findings.

**Abstract:**

The health monitoring of laboratory rodents is essential for ensuring animal health and standardization in biomedical research. Progress in housing, gnotobiotic derivation, and hygienic monitoring programs led to enormous improvement of the microbiological quality of laboratory animals. While traditional health monitoring and pathogen detection methods still serve as powerful tools for the diagnostics of common animal diseases, molecular methods develop rapidly and not only improve test sensitivities but also allow high throughput analyses of various sample types. Concurrently, to the progress in pathogen detection and elimination, the research community becomes increasingly aware of the striking influence of microbiome compositions in laboratory animals, affecting disease phenotypes and the scientific value of research data. As repeated re-derivation cycles and strict barrier husbandry of laboratory rodents resulted in a limited diversity of the animals’ gut microbiome, future monitoring approaches will have to reform—aiming at enhancing the validity of animal experiments. This review will recapitulate common health monitoring concepts and, moreover, outline strategies and measures on coping with microbiome variation in order to increase reproducibility, replicability and generalizability.

## 1. Introduction

The health monitoring (HM) of laboratory rodents builds the solid foundation for preserving animal health and ensuring the validity of biomedical research data. During the last century, enormous progress has been made regarding animal housing and gnotobiotic derivation processes, aiming at improving the microbiological quality of rodents bred in laboratory research facilities. Revisiting the last 140 years, the hygienic status of animals used for scientific purposes evolved from an early phase of domestication (~1880–1950), in which breeding stocks were established with animals naturally infected with diverse pathogens, over extensive gnotobiotic rederivation processes (~1960–1985), to a period where indigenous viruses and other specified pathogens were eliminated from laboratory mouse and rat colonies (~1980–1996). This roughly divided timeline, already chronicled by Steven H. Weisbroth and David G. Baker in the late 20th century [1,2], can be extended to the most recent and still ongoing situation, the phase of an isolated animal husbandry with limited microbial exposure. Though this development follows an intrinsic logical path, it turns out that the current situation also brings its own challenges. 

Nowadays, widely used hygienic monitoring programs and sanitation procedures lead to enormous improvement of the microbiological quality of laboratory animals, producing breeding colonies, which are free of pathogens and even free of most opportunistic pathogens [3,4]. Together with strict barrier housing in commercial vendor and experimental user facilities, this allows for efficient control of infectious disease development. Undoubtedly, modern rodent research management has improved the overall health status of colonies, in line with the 3Rs [5], and enabled the breeding of even severely immunocompromised animal models without microbial-induced diseases. Importantly, as functions of genes and noncoding genome regions are still largely unknown, the vast and even rapidly growing production of genetically altered animals results in a yet unknown number of mutants with immune defects, making strict barrier housing inevitable.

However, this concept of increased isolation also shaped a limited diversity of the animals’ gut microbiome, which dramatically differs between distinct animal facilities [6,7,8]. Rapidly developing molecular methods, allowing for high throughput analyses of various sample types, have revealed a striking influence of microbiome compositions on animal models, e.g., influencing immune system maturation and disease phenotypes and therefore the validity of biomedical research [9,10,11,12]. Scientists have noted this phenomenon and developed ideas to overcome this situation, especially in fields where “too clean mice” do not produce scientifically valid results [13]. These approaches range from the use of pet-shop mice to intentionally rewilding research models (see below). 

At a first glance, there is an obvious dilemma of two divergent goals, which both are fundamental in animal-based research. On the one hand, there is the desired goal to maintain standardized research animals infectious-disease-free, following the basic principle of animal welfare with the 3Rs as its foundation. On the other hand, scientists aim to produce findings that can be reproduced, replicated and finally translated to the human situation, following the principle of ensuring scientific value. The latter has recently been laid down in the concept of the 6Rs, which takes both animal welfare and scientific value into consideration [14]. In this review, we will outline both sides of this dilemma and describe current concepts and ideas regarding how to deal with this situation. All in all, these factors need to be respected to avoid loss of animal welfare or loss of scientific validity, both being indispensable in biomedical research.

## 2. Scope of Health Monitoring Management

**Figure 1:**
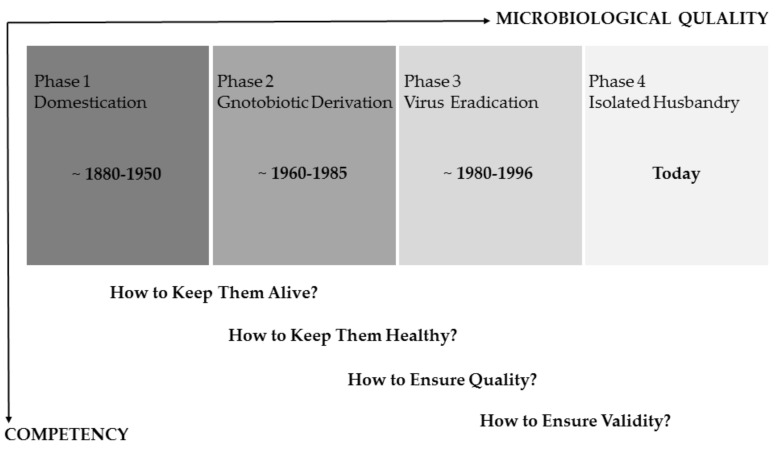
Schematic timeline roughly dividing the last 140 years of experimental rodent work into four different phases. The main scopes of health monitoring concepts evolved analogous to the improvement of the microbiological quality of laboratory rodents. The underlying questions reflect the competency necessary to implement/realize these concepts (modified from [1,2]).

As the microbiological quality of laboratory animals did evolve over time, so did the scopes of HM concepts (Figure 1). In the early phase of rodent maintenance, naturally infected breeding stocks potentially harbored various pathogens, causing severe clinical diseases and sudden deaths of whole animal cohorts. During this time, the main task was simply to keep live animals and, later, to identify causative agents, giving rise to the question: how can one keep them alive? With the help of gnotobiotic rederivation procedures carried out by hysterectomy or embryo transfer of the offsprings from infected donor animals and subsequent rearing by uninfected foster dams, typical pathogens were removed from colonies, redirecting to the question: how can one keep them healthy? [1,2]. With the development of reliable and more sensitive diagnostic (screening) methods, the scientific community became more and more aware that even subclinical infections with certain pathogens have a pronounced impact on different animal models, shifting the focus of HM towards aspects of research quality (how can one ensure quality?). Concrete recommendations for systematic HM programs [15,16,17] aimed at a high level of hygienic standardization by defining specific pathogens, which should be included in the HM procedure. This “inclusion” of microbial agents in the procedure often shaped the view of a maximal “exclusion” from the colonies, and repeated re-derivation cycles led to a situation that some rodents harbor an artificially limited microbiome [8,13]. Further improvement of diagnostic methods and the rapid development of molecular techniques currently create increasing awareness of the striking influence of the microbiome composition on animal models [9,10]. Modern HM concepts have to take this impact into account to ensure the value of biomedical science (how can one ensure validity?). This requires, among others, the inclusion of metagenomics techniques as well as a differentiated view on hygienic management.

In principle, all these historical stages still need to be respected in modern health-monitoring programs, as the impacts of pathogens on the one hand and the microbiome on the other hand are equally important. Therefore, responsible personnel needs to be proficient in the underlying competencies and skills, ranging from knowledge of microbial agents critical for animal’s health or research, necropsy and diagnostic techniques, basic understanding in epidemiology, up to the impact of the microbiome on research models.

## 3. How to Keep Them Alive

Before rederivation techniques became available, laboratory rodents were naturally infected with various pathogens, affecting their overall health status. Deaths due to infectious diseases became rare with improvement in husbandry techniques and declining prevalence of agents. However, enzootic outbreaks still pose a risk to experimental and breeding colonies, as barrier leaks can never be completely excluded, live animals are transported internationally for exchange, and immune-compromised animals are highly susceptible even to prevalent opportunistic pathogens. Accidental pathogen introduction also occurs via biological material, which is injected or otherwise intentionally applied to animals during experiments.

### 3.1. Microbial Agents Compromising the Animal´s Life

Classically, the sudden death of larger animal cohorts were to be expected in scenarios of an (acute) epizootic infection with viral agents, such as the *Murine Respirovirus*, also known as *Sendai Virus* (SeV), *Lymphocytic Choriomeningitis Virus* (LCMV), enterotropic variants of *Mouse Hepatitis virus* (MHV), or *Ectromelia Virus*. The last two viruses only infect mouse colonies. In contrast, SeV and LCMV have a broader host spectrum, causing disease in most rodent species and—in the case of LCMV—can also be transmitted to primates, including humans [1,4,18,19,20]. While most bacterial infections do not result in acute mortality, infection with *Clostridium* (*Cl.*) *piliforme*, the causative agent of Tyzzer’s disease, may lead to the sudden death of young animals, especially in the phase of weaning [1,4,21,22]. As an endo-spore-forming bacterium, *Cl. piliforme* is infective for long time periods in the environment, which—together with the ability to infect most mammalian species—caused high prevalences in laboratory rodent colonies [23,24,25]. Since Mongolian Gerbils are highly susceptible for Tyzzer’s disease, even infections of adult animals cause severe clinical symptoms in this species [18]. Fatal bacterial pneumoniae is one common cause of seasonal occurring acute death in laboratory guinea pigs. *Bordetella bronchiseptica* can frequently be isolated from infected lung or tracheal tissue often co-associated with other opportunistic pathogens such as *Pasteurella multocida* or *Streptococcus pneumoniae* [26]. Considering that mouse and rat colonies are very often subclinically infected with the aforementioned bacteria, different animal species should be housed separately in individual hygienic units within a facility.

### 3.2. Diagnostic Measures to Enable Disease Control

For effective disease control in animal facilities, clinical examination and necropsy of sick or deceased animals should be regularly performed by veterinarians, which are competent in basic diagnostic methods. Patho-histological evaluation of organ morphology serves as a powerful tool to identify common disease phenotypes and discriminate those likely caused by infectious etiology from typical genetically or environmentally induced disorders, such as hydronephrosis, ulcerative dermatitis or the lactation associated ileus [18,19]. Therefore, the training of staff in basic diagnostic skills and the possibility to perform on-site examinations is essential and should be the substantial part of every solid health monitoring program.

Particular attention needs to be given to the use of biological materials in in vivo research, as agents endangering animals’ lives and even zoonoses can be transferred into research vivaria. Collaborating researchers might share infected tissues and so involuntarily contribute to the spread of agents between facilities all over the world. Two different surveys systematically analyzed biological material (in this case: tumors) in the USA and Europe and consistently found that more than 50% of all analyzed mouse tumors harbored pathogens, mostly viruses, but also mycoplasmas and protozoa [27,28,29]. Actual data suggest that the prevalence is decreasing [30]; however, material might still remain in freezers and infections due to contaminated material still occur. Several outbreaks of Mousepox were reported to be caused by the introduction of the *Ectromelia Virus* to the Naval Medical Research Institute and Cornell University in the USA via contaminated, commercially purchased serum [31,32]. Unfortunately, the same scenario repeatedly happened a few years later, pointing out that the prospective screening of all biological material, which is to be introduced into an animal, is indispensable [33]. Besides the risk of causing severe disease in animals, there are also major human health concerns, as there are several reports of accidental infection of facility research employees with zoonotic agents such as Hantaviruses and LCMV due to the handling of subclinically infected research animals [34,35,36]. Traditionally, biological material can be screened by the use of so-called antibody production tests (mouse/MAP or rat/RAP tests) which are based on serological analysis of animals, several weeks after they were injected with the respective biological material [37,38,39]. As this in vivo testing strategy not only requires the use of animals but may also potentially fail since it relies on a complete immune response of injected individuals [33], it is nowadays nearly completely replaced by direct analysis of the biological material using Polymerase Chain Reaction (PCR) assays for defined pathogens [40,41,42].

## 4. How to Keep Them Healthy

Very few pathogens come along with high mortality rates in immunocompetent animals, but may cause clinical forms of disease, with symptoms exerting when individuals are susceptible to an infection with the respective pathogens. Nowadays, a bewildering number of mouse and rat models are available from a wide variety of commercial and non-commercial sources. Due to advances in transgenic techniques and especially the development of genome editing technologies [43,44], their number is constantly increasing. These models can substantially differ in their immunological response according to their genetic background and/or their genetic alterations and therefore may show different symptoms and vary in the severity of disease development after infection [45,46,47,48,49,50].

In fact, most rodents experience subclinical forms of infection, meaning that they become a carrier of pathogens, without developing clinical symptoms. When subclinical infected animals remain undiagnosed, they may uncontrollably shed infectious agents in the environment and infect other individuals of the colony. The infection may then become enzootic, resulting in pathogen persistence. The introduction of new strains to the animal room will then lead to an epizootic infection of the naïve animals, causing more or less severe forms of diseases, depending on their susceptibility to the respective pathogen.

### 4.1. Agents Affecting the Animal’s Health Status

Most infectious agents are causing the most pronounced symptoms in very young animals, typically in the phase of weaning, when other stressors and the absence of maternal antibodies allow uncontrolled pathogen proliferation [1,4]. A classic example of age-dependent disease development is an infection with Rotaviruses, resulting in the epizootic diarrhea of infant mice (EDIM), which is caused by the Mouse Rotavirus Type A, or the infectious diarrhea of infant rats (IDIR), caused by the Rat Rotavirus Type B [51,52,53,54,55]. Although animals of all age groups are susceptible, clinical disease in the form of watery stool, lethargy and distended abdomens exclusively occurs in animals infected between birth and about two weeks of age, which differentiates EDIM/IDIM from other infections associated with diarrhea, such as Tyzzer’s Disease or enterotropic forms of MHV. Those substantial age-dependent differences regarding virus susceptibility are major advantages in terms of sanitation procedures, as virus eradication may be relatively simply achieved by a prolonged breeding cessation and strict hygienic measurements, as it was successfully conducted after an EDIM outbreak within an experimental barrier housing mice [56]. Due to sanitation procedures and strict hygienic measures, the actual prevalence of mouse and rat Rotaviruses is nowadays low; however, outbreaks occasionally occur, as was recently reported after exposure of exported mice to contaminated shipping boxes [57].

Generally, most rodents are relatively prone to develop chronic respiratory diseases. Rats are especially susceptible to infections with *Filobacterium rodentium*, formerly known as *Cilia-associated respiratory* (CAR) *Bacillus* or *Mycoplasma pulmonis*, which both can cause severe airway infection, particularly when animals are predisposed due to poor husbandry conditions or co-infections with other (opportunistic) pathogens [58,59,60,61,62,63,64]. Since viral pathogens were mostly eradicated from laboratory rodent colonies, opportunistic bacteria are probably the most common threat to the animal’s health status. In this context, members of the family *Pasteurellaceae* can be considered as classical pathobionts, since they are often part of the normal flora of mucosal sites, but may also induce inflammatory conditions such as conjunctivitis, dacryoadenitis or multiple abscess formations in susceptible mouse and rat strains [65,66,67,68,69,70]. Recently, rodent *Pasteurellaceae* were reclassified into the novel genus *Rodentibacter*, with *Rodentibacter pneumotropicus* and *R. heylii* (formerly known as [*Pasteurella*] *pneumotropica*) as the most prevalent species in laboratory mouse and rat colonies [71]. *Staphylococcus aureus* and *Klebsiella* sp. are also highly prevalent opportunists in laboratory rodents and as both are zoonotic agents, infection may be transmitted from animals to humans—and also the other way round. However, two comprehensive studies predominantly found host-adapted *Staphylococcus aureus* isolates in laboratory mouse and rat colonies, which were sensitive to Methicillin treatment [72,73]. Although both agents are not necessarily harmful to the animals, susceptible strains may develop clinical symptoms due to natural bacterial colonization. Development of otitis media caused by *Klebsiella oxytoca* was repeatedly described for C3H/HeJ mice, which express a dysfunctional Toll-like receptor 4 and therefore exhibit impaired innate immune mechanisms due to insufficient pathogen recognition [74,75]. Furthermore, *Klebsiella* induced urogenital infections and pneumonia in LEW.1AR1^iddm^ rats, which are prone to develop type1 diabetes mellitus mediated by autoreactive T-cell populations [74]. While in these cases disease development occurred in somehow immune-modulated animals, two independent studies described the development of *Staphylococcus aureus*-induced Botryomycosis and facial abscesses in susceptible mouse outbred strains [76,77].

Amongst that, some animals are highly susceptible to infections with *Helicobacter* sp. [78,79,80,81], e.g., immunodeficient mouse and rat strains suffer from severe intestinal inflammation. Here, especially *Helicobacter hepaticus* may also induce further pathologies such as hepatitis and inflammation-induced colon carcinogenesis, as Mangerich et al. described it after infection of *Rag2*-deficient mice [82]. Until now, at least nine distinct *Helicobacter* species (namely, *H. hepaticus, H. typhlonius, H. bilis, H. rodentium, H. ganmani, H. muridarum, H. mastomyrinus, H. rappini, H. trogontium*) were isolated from the gastrointestinal tract of laboratory rodents, which is why co-infections are not uncommon and may worsen disease development [83,84,85]. Since most genetically modified immunodeficient mouse and rat strains lack sufficient pathogen defense mechanisms, those animals are generally prone to develop infectious diseases and, as a result, unspecific intestinal inflammation may spontaneously occur after exposure to mixed opportunistic agents [86]. For the same reasons, immunodeficient rodents may develop other pathologies after infection with microorganisms, which are generally not harmful to immunocompetent animals. In this context, it was recently described that NSG and NRG mice developed ascending pyelonephritis after surgery, which could be followed up to an infection with *Candida albicans*, picked up after i.v. injection within a restrainment device [87]. Other reports are numerous and involve the development of hyperkeratotic dermatitis induced by *Corynebacterium bovis* [88,89], otitis media caused by *Ralstonia picketii* [90] or even the development of severe septicemia after bacterial translocation of commensal bacteria from the normal intestinal flora [91]. One of the greatest health threats of immunodeficient animals, when housed together with immunocompetent strains, is certainly respiratory disease and chronic wasting due to infection with *Pneumocystis* sp., a fungus causing severe interstitial pneumonia [92,93]. Although lung inflammation may occasionally also occur in immunocompetent animals [94], an intact adaptive immune response will normally prevent disease development. However, subclinically colonized animals may transmit infectious agents to susceptible hosts, leading to severe clinical symptoms in immunodeficient members of the colony [95], while co-infections with other opportunistic agents may worsen overall pathology [96,97].

### 4.2. Hygienic Measures to Maintain the Health Status of Laboratory Rodents

Today it is standard that laboratory rodents and especially all animals with impaired immune functions are housed within strict barrier systems, preserving a protective biocontainment, which allows for breeding healthy animals. It is of major importance to establish routine screening programs, aiming at pathogen detection at an early time point of infection. Here, the use of traditional bacteriological, parasitological and virological diagnostics methods in combination with both prospective and indicative sampling procedures allows for routine pathogen screening as well as clearing up observed abnormalities within a colony and detection of causal infectious agents. In this context, regularly performed necropsy of sick or deceased animals, together with classic patho-histological analyses, is of major importance, as it is the fundament for the discovery of novel pathogens, especially viral agents, which otherwise will remain unidentified. Based on this, the *Murine Norovirus* (MNV) as well as the *Mouse Kidney Parvovirus* (MKPV), which was discovered recently as the causative agents for inclusion body nephropathy in immunodeficient mouse strains, could be identified as novel murine viruses [98,99]. Since it has been unknown and therefore not included in routine diagnostics for a long time, prevalence of MNV is typically high in the facilities [100,101,102,103,104,105], and colonies may yet have to be re-derived by hygienic sanitation procedures to establish a virus-free status.

## 5. How to Ensure Quality

Beside the sheer intention to improve the overall health condition of laboratory animals, it is also of major importance to standardize the hygienic conditions within animal experiments to ensure the quality of biomedical research data. The number of publications reporting the influence of infectious agents on experimental results is countless, which gets even more problematic in subclinical infected animals when the infection remains undiagnosed. Therefore, standardized and up-to-date health monitoring strategies need to be in place at each animal facility to ensure all of the following: life, health and quality.

### 5.1. Microbial Agents Impacting “Quality”

Infections with mouse (Mouse Parvovirus (MPV1–5), Minute Virus of Mice (MMV)) and rat (Kilham Rat Virus (KRV), Rat Minute Virus (RMV), Rat Parvovirus (RPV), Toolan’s H–1 Virus) parvoviruses may be one of the most prevalent but also frequently underestimated natural viral contaminations of laboratory mice and rat colonies. As most *Parvoviridae*, rodent parvoviruses require actively dividing or differentiating cells for survival and therefore predominantly replicate in endothelial cells, lymphocytes and hematopoietic precursors [1,49,106,107]. Although natural infections usually remain asymptomatic, there are several reports of reproductive abnormalities, when infection occurred during fetal development [108]. Furthermore, experimental infections with MVM led to myelosuppressive and lysis of T–Lymphocytes in neonatal and immunodeficient animals, which even became lethal dependent on the administered virus dose [109,110,111]. Likewise, Toolan’s H–1 Virus was found to experimentally induce CNS malformations and deformations of the skeletal system as well as increased hepatocellular necrosis after provoked liver injury [1].

Despite those clinical manifestations, the impact of parvovirus infections on research results is huge, as it will massively influence processes linked to cell proliferation. While some authors observed immunomodulatory and myelosuppressive properties [112,113,114], mouse and rat parvoviruses also reveal a strong oncosupressive potential, leading to decreased growth rate and rejection of tumor cells [107,115,116]). Since parvoviruses may cause persistent infections of a colony, researchers should be aware of the parvovirus status of their animals and the striking influence that may have on their results, especially in the field of immunology and oncology. However, elimination of these agents from an infected colony may be challenging, due to its high environmental tenacity, residual risk of infection after hygienic re-derivation procedures and difficulties in diagnostics [107,117,118]. Mouse-and-rat-strain-dependent seroconversion and intermittency of viral shedding result in seemingly low prevalences within a given colony, which requires testing of quite large animal numbers to successfully reveal ongoing infections [107,117,119,120,121]. However, health surveys from all around the globe showed that seroprevalence of parvoviruses in laboratory rodents as well as in pet-shop animals is generally high, indicating that infections remain endemically persistent in most facilities [100,101,102,103,104,105]. The most prevalent virus within mouse colonies is the *Murine Norovirus* (MNV) [100,101,102,103,104,105], probably due to its relatively recent discovery in 2003 [98]. Until then, MNV infections remained undiagnosed and therefore mostly unaffected by rederivation processes. Generally, MNV infections are asymptomatic in immunocompetent mouse strains and clinical forms of disease are very rare, even in immunodeficient animals [122]. However, MNV infection can lead to gastrointestinal pathology in susceptible mouse models [123,124], e.g., MNV causes intestinal epithelial barrier disruption in *Interleukin-10*-deficient mouse strains, which spontaneously develop chronic typhlocolitis depending on microbiological stimuli and are commonly used as a murine model for inflammatory bowel disease. In a comprehensive study using a gnotobiotic mouse model, Basic et al. demonstrated that MNV exacerbated the inflammatory phenotype, serving as a potent colitogenic stimulus, pointing out its possible impact on gastrointestinal research data [124,125]. Therefore, it is inevitable that scientists are aware of the potential impact of certain agents on their specific field and consider them in the hygienic status of their colony.

Within the group of bacteria, *Helicobacter* sp., *Staphylococcus aureus* and *Rodentibacter* spp. are the most prevalent agents in laboratory rodent colonies, which not only affect animal health by causing opportunistic infections in susceptible strains, but may also interfere with research results in the case of subclinical forms of disease [126,127,128]. Parasitic infections are also common in laboratory rodents, mostly including endoparasites such as pinworms or protozoa [101,102,105]. Other agents such as *Filobacterium rodentium* and *Mycoplasma pulmonis* are less frequently found but may strongly interfere with immune responses, if present in the colony [129,130,131,132]. Concerns should generally come up regarding zoonotic agents, which are nowadays rarely, but still occasionally, detected in laboratory and also pet-shop rodents [133,134,135]. Actual infections of humans regularly occur, especially in people involved in the care and husbandry of subclinically infected animals [135,136,137] or after injury with contaminated material [138]. In this context, Hantaviruses may be most concerning, as acute infections are a serious threat for human health. A systematic literature survey, based on the serology of staff, determined that 180 persons suffered from clinical disease manifestation and 85 persons were subclinically infected and unknowingly acquired Hantavirus infections from working with rat colonies [137]. The same study investigated the prevalence of infections with LCMV and concluded that thousands of employees and pet-shop customers were probably unaware of their potentially exposure to infected animals due to a major LCMV outbreak in 2012 in the United States of America. Special attention should be paid to the fact that infection with both agents may be airborne, which makes protective measures indispensable, especially when persons are exposed to excretions of rodents. Infections with *Streptobacillus moniliformis*, the causative agent of the so-called “rat bite fever” are also relevant but can be diagnosed more easily, as most infections involve positive history of a rat bite [139,140].

### 5.2. Measures to Ensure Quality—Standardized Health Monitoring

The Federation of European Laboratory Animal Science Associations (FELASA) has published a number of recommendations for the health monitoring of animals used for scientific purpose. Those recommendations are regularly updated and available for different species, including rodents, guinea pigs and rabbits [17], ruminants and pigs [141] and non-human primates [142]. The first publication for rodents was released in 1994 [15] with the primary intention of harmonizing the health monitoring procedures between animal facilities, especially to facilitate the exchange of animals between cooperating researchers. As knowledge about infectious agents increases over time, those recommendations were yet revised twice [16], with the last version published in 2014 by Mähler et al. [17]. Here, quality criteria are defined, which serve as a valuable orientation for systematic HM programs in facilities assigned with the breeding or experimental use of laboratory animals, facilitating processes harmonization and reporting of the hygienic status. According to those recommendations, the main goals of an HM program should include overall health improvement (exclude clinical infections), enhancement of general biosafety (exclude zoonotic infections) as well as prevention of interference of the hygienic status with research results (exclude subclinical infections). Therefore, this publication provides lists of relevant viruses, bacteria, fungi and parasites (choice of agents) including concrete recommendations for their test frequencies, depending on the respective risk of infecting the colony. Furthermore, commonly used test methods, selection of animals/sentinels and results interpretation are critically discussed, aiming to improve the reporting of the hygienic status and the writing of appropriate health certificates. A colony monitored according to these recommendations can be referred to as “specified pathogen free” (SPF). Unfortunately, it became more and more common to misinterpret the intention of this publication and to use it as a simple exclusion list. Contrarily, the authors intended to contribute compact information of current knowledge in laboratory animal medicine and emphasize that HM programs should be complexly designed and supervised by a competent person, educated in the field of infectious biology in veterinary medicine.

The most critical part of a systematic HM approach is probably the choice of agents, which should be primarily defined by their zoonotic potential, pathogenicity (obligate pathogens, opportunists, and pathobionts) and possible impact on results. The abovementioned recommendations serve here as a valuable tool, since they consider relevant agents on their individual risk. Hence, frequently found pathogens such as parvoviruses or MHV should be monitored at least quarterly, whereas an annual monitoring frequency should be sufficient for less prevalent agents, such as LCMV or Ectromelia. In addition to those obligatory agents, Mähler et al. also defined some additional agents, which do not necessarily have to be included in an HM program, depending on their individual relevance to the facility. Most importantly, the authors also pointed out that the panel of monitored agents has to be critically adapted to the current situation and that more agents should be included, if considered relevant. Therefore, the provided list should not be uncritically interpreted as carved in stone, but rather used as a solid foundation to work on.

Another essential a priori consideration is the appropriate size of a colony, for which the HM results have to be summarized. Therefore, hygienic units should be defined, which are characterized as those parts of a facility in which animals, personnel and material are transferred freely—without hygienic measures—and which can consequently be assumed to harbor the same germ spectrum. The size of such a hygienic unit can vary immensely, dependent on the applied hygienic measures. It can simply be a single animal room if animals are housed in open cage systems and every room has its individual equipment. However, it can also be a whole floor, consisting of several animal rooms, if personnel is allowed to switch between rooms without changing the protective clothing. The minimum size may be even a single cage, if animals are kept within micro-isolation conditions, as they exist in IVC housing systems (appropriate handling using a laminar flow safety cabinet and disinfection procedures implied). IVC cage systems can effectively prevent transmission of infections within a colony, but also kind of complicate routine HM procedures as monitoring obviously cannot be conducted on cage-containment level. To solve this problem, traditional health monitoring concepts often make use of sentinel animals (see below). As this approach has its limitations and is not suitable for all agents, it is essential to include direct testing of colony animals, especially when they show clinical signs of disease, to enhance the diagnostic success for infectious agents.

### 5.3. Key Figures of Standard Health Monitoring

**Figure 2:**
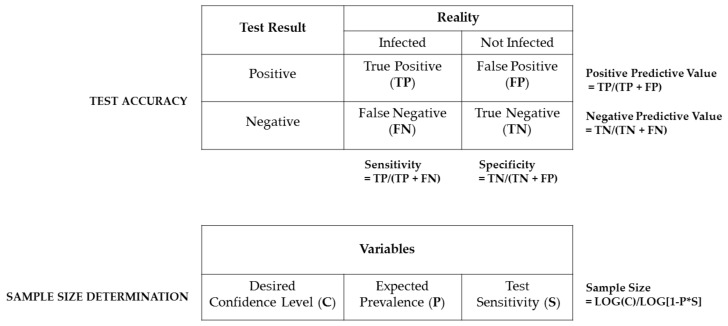
Schematic overview of the two main factors influencing the diagnostic success. The test accuracy is defined by its diagnostic sensitivity and specificity as well as the underlying positive and negative predictive values. All parameters can be calculated based on true positive (TP) and negative (TN) as well as on false positive (FP) and negative results (FN). The number of required samples necessary to detect an infectious agent is based on the desired confidence interval (C) expected pathogen prevalence (P) and the respective test sensitivity (S). Importantly, this calculation holds only true for colonies of at least 100 animals and free distribution of infections [17,143,144,145].

In general, two factors affect the overall diagnostic success (suitability of the chosen method implied): test accuracy and sample size. The accuracy of a diagnostic test can be described by its sensitivity and specificity (Figure 2), with the first one certainly more important for the probability of simply finding a certain infectious agent and the latter one defining the diagnostic relevance of the results. The higher the diagnostic sensitivity, the lower the amount of false negative results will be, keeping the number of under-diagnosed positive cases at a minimum. The diagnostic specificity will be higher the lower the amount of false positive results, keeping the number of over-diagnosed negative cases at a minimum. Both parameters are crucial for results interpretation, as they define the positive and negative predictive value of a test procedure.

The sample size determines the required number of animals, which have to be tested to reliably find a pathogen within a colony, and it can be statistically calculated. The underlying equation (as depicted in Figure 2) consists of the desired confidence interval (expressing the highest risk of a false negative result), the test sensitivity, the expected prevalence of the monitored pathogen as well as the size (number of animals) of the respective colony [17,143,144,145].

It is important to keep in mind that this equation is only valid in cases where infectious agents can randomly infect all animals within a husbandry unit, which is rather unlikely in modern Individually Ventilated Cage (IVC) housing conditions. Therefore, husbandry forms, which keep animals within cage-level containments, require alternatives to randomly choosing colony animals, which may be realized by using so-called sentinel animals.

Sentinels are intentionally exposed to infectious material, aiming at causing an infection with (all) the pathogens present in a husbandry. The HM of those sentinels may then representatively reveal the hygienic status of the colony. There are different types of sentinel systems, differing in the type of pathogen exposure. The most common approach is to expose the sentinels to the soiled bedding of the colony animals (soiled or “dirty” bedding sentinels), which was described as a suitable monitoring system for infectious agents which are shed into the animals’ feces, urine or other excretions [146,147,148,149,150,151,152]. To increase the pathogen load, unintentional “dilution” of infectious agents may be averted by housing the sentinel animals directly in the complete used cages of the colony, where they also get in contact with nesting material, food and water bottles. Although this approach requires a longer time period to monitor whole IVC rack systems, diagnostic success rate will probably be higher, as it was shown that critical agents such as *Rodentibacter* spp. were detectable using this method [153] However, independent of the concrete variant, bedding sentinels are not suitable for the reliable detection of ectoparasites (e.g., fur mites) or pathogens, which are solely transmitted via aerosols (e.g., SeV) [148,154,155,156]. In those cases, other sentinel types (direct contact sentinels or exhaust air dust sentinels), direct testing of colony animals or environmental testing approaches (see below) are obligatory.

### 5.4. Recent Developments in Standard Health Monitoring

Over the last decades, the use of PCR and, in the case of RNA viruses, Reverse Transcription (RT)–PCR techniques have been broadly validated as a valuable alternative to traditional cultural, microscopic and serological pathogen detection methods. As PCR testing is based on the molecular detection of specific genetic sequences of the infectious agents, this method can be used to detect even very small amounts of nucleic acids in various sample types, which not only enhances the diagnostic sensitivity but also facilitates the use of environmental sample material as an alternative to direct animal testing [157]. Since infected animals shed the infectious agents via multiple routes (feces, urine, saliva, other excretions, skin/fur…) in their home – cage environment, this material can be analyzed instead of testing samples directly obtained from the animals. Depending on the respective pathogens, different sample types can be used, either at single cage level (bedding and/or nesting material, dried fecal pellets, cage swabs or filter material from cage lids) or also at rack level. An elegant solution, especially in terms of screening larger animal cohorts, is the analysis of exhaust air dust (EAD) material, which can be also sampled at cage level but also for a whole IVC rack system, depending on the location of air filtration procedure [158]. In case of air filtration on the rack level, samples can be obtained by directly swabbing the exhaust plenum or analyzing pre-filter material of the exhaust air stream. For the latter method, different commercial systems are available which are more or less equally efficient for PCR-based pathogen detection methods [159,160]. However, there are also reports of successful implementation of alternative techniques using gauze material, manually placed on the prefilters or cage lids [161,162,163] or the direct analysis of the filter top material [164].

There are increasing numbers of publications, which describe that the analysis of environmental sample material, and here especially the use of EAD, is superior compared to traditional sentinel systems and more and more facilities report that they have completely replaced the use of sentinel animals by implementing EAD testing [160,165,166]. This paradigm shift is particularly in line with the 3Rs, as most classical HM programs involve scarifying sentinel mice (depending on the sample types), while environmental sampling can lead to a reduction of animals used for HM programs or even replace them totally. Additionally, there is common evidence that the PCR-based screening of environmental sample material can significantly increase the diagnostic success of the monitoring procedure, which can further reduce the required number of animals for experiments, as this significantly improves their hygienic standardization. Finally, environmental sampling techniques may also refine animal experimentation in terms of an overall health improvement of the colony due to the prevention of infectious diseases.

Until now, EAD-based monitoring approaches proved to be suitable for a broad variety of infectious agents, such as MNV [161,167], MHV [158], *Murine Astrovirus* [168], *Lactate–Dehydrogenase–Elevating–Virus* (LDEV) [169], *Rodentibacter* sp. [153,162], *Helicobacter* sp. [158,159,167], *Mycoplasma* sp. [160], *Pneumocystis* sp. [160], fur mites [158,168,170], pinworms [158,164,171] and enteric protozoa [158,159,160]. However, infections with murine Parvoviruses and *Klebsiella oxytoca* could not be confirmed by this method so far [158,159], and in the case of *Staphylococcus aureus* there exist contrasting results [159,160]. Future studies are necessary to close this gap of knowledge and validate the suitability of EAD-based testing for all pathogens relevant for laboratory rodents, especially for those with lower shedding concentrations, such as parvoviruses.

The major advantage of PCR-based testings—enhanced method sensitivity and a marked reduction of false negative test results—may also be one of its drawbacks, as sample contamination can easily occur [157]. False positive results can have dramatic effects in terms of consequences due to hygienic decision making (e.g., depopulation of a whole colony), which is why a single positive PCR result should always lead to subsequent confirmation of an infection. This is especially important, since the molecular detection of nucleic acids of pathogens does not automatically imply that the colony is indeed infected, for the reason that this method cannot distinguish between living and dead organisms and positive results may still occur after the detection of residual DNA/RNA present in the sample material. Residual nucleic acids may still even be present after common decontamination procedures such as autoclavation or irradiation, which is why washing procedures of cages and whole IVC rack systems will certainly play an essential role for preventing concurrent misidentification. Generally overcoming this, positive results should always be confirmed using traditional detection methods, e.g., serology, to make sure that the colony is truly infected.

Besides the risk of false positive results, false negative results may also occur, especially when the primers used for gene amplification are not suitable to detect all relevant (sub)strains of an agent. Thus, a PCR assay can only be as good as its molecular design, which mainly depends on the accuracy and actuality of underlying databases. Particularly, rapidly evolving pathogens frequently gain point mutations within their genomes, which may lead to the failure of primer binding and henceforth to false negative results [157]. Therefore, the genetic drift of agents has to be taken into account and assays used have to be permanently adapted to the current research.

Moreover, PCR only detects defined genetic sequences, whereas the use of culture or microscopic methods can expose the entirety of (cultivable) agents present in the sample material and reveal dynamic changes within a colony. This is of great value as this might point towards a leak within a barrier, which requires immediate initiation of preventive measures.

Budget may also play a role, as molecular detection methods can get quite cost intensive, especially when sample analysis is performed by commercial laboratories. However, there are reports that PCR-based testing of EAD material is actually less expensive as the traditional sentinel approach, mostly due to lower costs for animal husbandry [172]. Altogether, the use of environmental sample PCR testing material is beneficial in modern HM concepts and should be used as a valuable tool, supplementing basic diagnostic methods, particularly in terms of animal welfare.

## 6. How to Ensure Validity

In general, animal studies need to be reproducible, replicable, and generalizable to ensure translational value of the respective research. As the microbial status of research animals might display a major confounder impacting study results, it needs to be controlled. While routine HM programs, as recommended by the FELASA, include screening for typical pathogens, there is clear evidence that other agents as well as microbiome composition or the microbial history of an animal may also influence distinct phenotypes, dependent on the animal model. Knowledge about potential impacts is essential to ensure the validity of research data; however, the causative agents are often not part of the routine HM program of facilities. Therefore, control of these agents or the microbiome of experimental groups has currently to be explicitly requested or self-conducted by responsible investigators.

### 6.1. Microbial Impact on Model Phenotypes and Research Validity

Some agents, which colonize the mucosal surfaces of animals as typical commensals, are considered pathobiontic and exacerbate certain disease phenotype or even may also independently induce pathologies in susceptible strains. In this context, *Proteus mirabilis* was shown to induce damage of dopaminergic neurons and motor functions in mice, hence strongly influencing models of Parkinson’s Disease [173]. Likewise, germfree *Interleukin-10*-deficient animals developed intestinal inflammation after mono-colonization with *Enterococcus faecalis*, a common intestinal microorganism of animals and humans [174]. Comprehensive lists of other reported examples were reviewed elsewhere [10,11,12,175,176] and should be critically accessed in the phase of project planning to take possible impact into account and/or implement novel screening procedures of breeding colonies or experimental animal cohorts.

With the expanding utilization of high throughput molecular screening methods, such as the 16S rRNA gene sequencing analysis or other metagenomic techniques, the amount of research demonstrating the striking influence of common commensals on animal models is rapidly increasing. Since some agents have detrimental effects on disease phenotypes [177] and therefore enhance disease susceptibility, others could be identified as protective factors, improving certain immune functions. Here, especially *Akkermansia muciniphila* was shown to confound immunological research data, as its abundance controls diet-induced obesity in leptin-deficient mice [178] as well as islet autoimmunity in the NOD model [179] protecting animals from diabetic phenotypes. On the other hand, *Akkermansia muciniphila* exacerbated *Salmonella-typhimurium*-induced gut inflammation in a gnotobiotic mouse model [180], whereas it protected mice from developing DSS-induced intestinal inflammation [181], revealing contrasting effects on colitis phenotypes. Similarly, *Segmented Filamentous Bacteria* (SFB) could be identified as protective factors during *Salmonella enteriditis* infection and were also shown to ameliorate intestinal inflammation in *Il10*-deficient gnotobiotic mice after co-infection with MNV and defined microbial consortia, dependent on their intestinal colonization ability [125,182]. However, it could also be shown that SFB induced intestinal inflammation in a T-cell transfer colitis model [183] and drive autoimmune arthritis, stimulating a Th17 driven immune response [184,185]. *Faecalibacterium prausnitzii* and *Bacteroides* sp. were also considered relevant, particularly in experimental colitis models, where they mainly protect animals from developing intestinal inflammation [186,187,188,189,190,191,192]. In an excellent review article, recently published by Hansen et al., relevant bacterial species and their potential impact on animal models were summarized, serving as a valuable basis for individual consideration and inclusion in HM protocols [175].

To understand the impact of commensals in animal models and to investigate their possible role in disease development, gnotobiology serves as a powerful instrument. After germfree rederivation using hysterectomy or embryo transfer under sterile conditions, animals can be associated with defined microorganisms to study complex microbial–host interactions during animal experiments [193].

However, influence on animal models is not always exclusively mediated by single agents, but influenced by completely microbial consortia, colonizing a different part of the animal. Especially the gastrointestinal mucosa is a major habitat for a manifold number of bacteria, protozoa, fungi, archaea and viruses, forming a complex community of more than 100 billion microorganisms per gram of intestinal content, also referred to as “gut microbiota” or the “microbiome”. These microorganisms are essential for the host’s physiological functions, such as the digestions of fibers and delivering vitamins, but can also be associated with the development of certain diseases [9,10]. Recent progress in using molecular techniques such as genetic sequencing and large-scale quantitative analyses, which can be referred to as microbiome analysis, reveals the striking influence of individual microbiota compositions on animal models and correlates those data with certain disease phenotypes. Since 16S rRNA-based sequencing approaches are restricted to the analysis of bacterial communities, so-called “Next generation Sequencing” techniques make use of the random amplification of shorter gene fragments (shotgun sequencing) and subsequent alignment using bioinformatic data bases, which facilitates the simultaneous detection of all types of infectious agents, also including viruses [157,194,195].

Intestinal immunology probably constitutes most of the research areas affected by microbiome compositions, as gut inflammation may be triggered by several microbiological factors. In this context, it is well known that *Interleukin-10*-deficient mice differ in the severity of colitis development, depending on the housing conditions and even when they are maintained at different institutions [196,197], and that composition of the gut microbiome is a primary determinant of disease severity [198]. This model could also be used to demonstrate the influence of different minimal bacterial consortia, namely, the Altered Schaedler Flora (ASF) and the so-called Oligo Mouse Microbiota^12^ (OMM^12^), on the level of intestinal inflammation after MNV triggered colitis induction [125], proving that disease severity depends on the microbial context. Other murine colitis models also depend on microbiota compositions, since colitis development after naïve T-cell transfer into immunodeficient recipients also heavily depends on the gut microbiome [199]. Furthermore, there are also several reports of the striking influence of the microbiota composition on phenotypes in colitis models using chemically induced intestinal inflammation [175,200]. Likewise, susceptibility to *Salmonella* infection is strongly influenced by endogenous *Enterobacteriaceae* [201], and intestinal epithelial adherence and the subsequent colonic survival of *Citrobacter rodentium*, a common model for infection with enteropathogenic and enterohemorrhagic *Escherichia coli* (EPEC and EHEC) in humans, completely rely on commensals [202], pointing out the strict dependency of infection experiments on the entire microbiota. Moreover, microbiome compositions also modulate colon tumorigeneses [203], diabetic phenotypes [204,205,206] and also impact models of liver disease, as comprehensively reviewed elsewhere [207,208].

**Figure 3:**
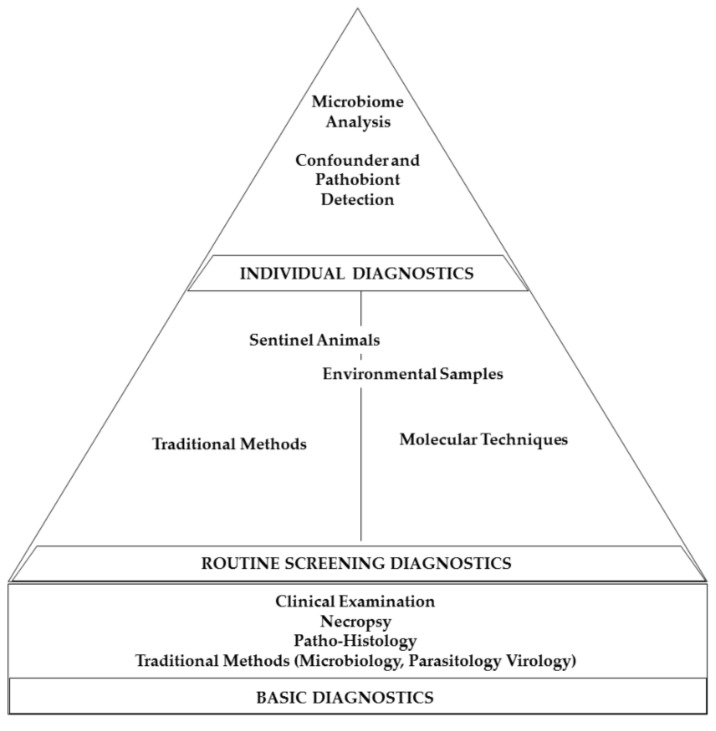
Diagram depicting the different proportions of HM programs. Basic diagnostic methods should build a solid foundation, and routine diagnostic screening procedures should form the main body of HM concepts, supplemented by individual diagnostic strategies.

Remarkably, besides gastrointestinal research areas, the animal’s microbiome also plays a significant role in neurological processes, influencing anxiety-related or autistic behaviors [209,210,211,212,213] and also the outcome of experimentally induced autoimmune encephalomyelitis [214]. Therefore, microbiome analysis or selective analyses of known study confounders should supplement every solid HM approach (Figure 3).

### 6.2. Measure to Ensure “Validity”

The benefit of repeated hygienic re-derivation procedures and strict barrier husbandry facilitated pathogen exclusion, aiming at improving the overall animal health and increasing the level of hygienic standardization. However, isolated husbandry also shaped a limited diversity of the gut microbiome. This resulted in significant variation of gut microbiota compositions, not only between distinct animal facilities, but possibly also within a facility, when strains are individually housed in different barriers or biocompartments [8]. This unintentionally induces a “secondary” lack of microbiological standardization, which may play a crucial role in the increasing number of reports of research data that cannot be replicated when conducted by different research groups, currently referred to as the so-called “reproducibility crisis” [215,216,217,218]. A solution is to enhance the level of microbiome standardization (see Section 6.2.1), which will improve the ability of researchers to reproduce findings of animal studies and which is therefore a measure to ensure the internal validity.

**Figure 4:**
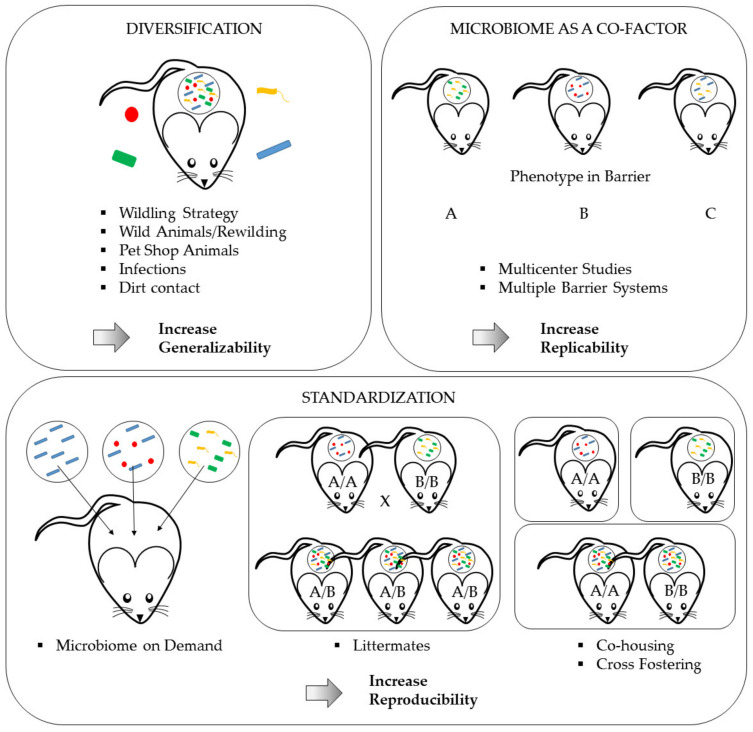
Measures to ensure research validity in the microbiome era. Intentional microbiome diversification will increase the overall generalizability of study results. Using the microbiome as a co-factor by comparing phenotypes from studies under different barrier conditions will uncover microbiome impacts on phenotypes. Thus, this method will identify stable phenotypes and therefore increase the replicability of findings. Finally, achieving a high level of microbiome standardization will markedly improve study reproducibility.

**Figure 5:**
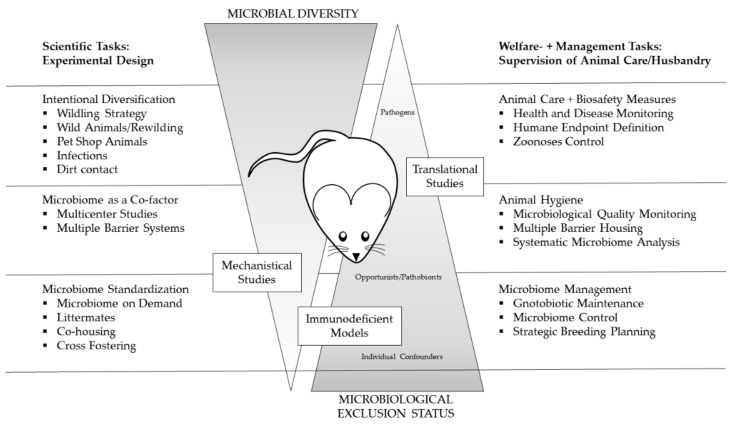
Dependencies of scientific strategies to cope with microbiome challenges, animal´s hygiene status as well as welfare and management tasks. The primarily scientific tasks regarding study design (depicted on the left-hand side) have enormous implications on the scope of hygienic management and health monitoring as well as on the veterinary care and colony management (depicted on the right hand side). Depending on the study planned, the microbial diversity and microbiological exclusion status have to match the requirements of the different animal models to ensure both scientific value and animal welfare, as implied by the 6Rs [14].

However, there is increasing concern that those findings will only be true for those exact study conditions, which will potentially decrease the generalizability of the results. In this context, Wuerbel et al. proposed to intentionally introduce a certain level of controlled variation, to test a phenotype for its robustness against natural confounders and therefore increase the translational value for further clinical studies [219]. An excellent review published by Witjes et al. discussed the pros and cons of standardization in the context of microbiome variation and concluded that standardization has to be increased when researchers conduct fundamental studies, especially those who aim at identifying disease mechanisms, to reduce study confounders, whereas more translational studies require robustness tests using mice with different microbiomes to ensure generalization and to increase the external validity of study results [220]. Possible solutions for inducing variation are either the intentional diversification of the microbiome using rather extreme measures such as the re-wilding of laboratory rodents (see Section 6.2.2) or using the microbiome as a co-factor in scientific studies (see Section 6.2.3), e.g., in multi-center studies. The abovementioned measures are summarized in Figure 4. Though these are primarily scientific tasks impacting the experimental design, they have enormous implication on the scope of hygienic management and health monitoring as well as on the veterinary care and colony management in animal-based research (Figure 5).

#### 6.2.1. Standardizing the Microbiome

Since reproducibility is closely related to research validity, it can be seen as a measure for the generalizability and translational value of results [217], thus determining relevance of findings [218]. Re-enhancing the level of microbiological standardization increases the so-called “internal validity”, which can be achieved by accounting for reciprocal host–microbiome interactions aiming at minimizing variation due to microbiological confounders [220,221]. However, the factors influencing the animal’s microbiome are countless and range from housing-related factors such as food, water, caging systems, bedding material (and of course their decontaminative treatments such as autoclavation, irradiation or acidification) to host-related factors such as the animal’s genetics, age and gender. Moreover, also infections with primary pathogens or psychological stress leads to an enormous variation of the fecal microbiome, shaping a massive microbiome variability between different animal vendor facilities [6,7,8,222,223]. In an excellent review article written in 2017 by Franklin and Ericsson, different sources of gut microbiome variability are summarized and measures are discussed to address this variability for improving research reproducibility [215]. These factors will make it virtually impossible to exactly adjust the microbiome of several breeders, facilities and even barriers. However, within barriers and even within studies, standardization of the microbiome is achievable.

Microbiome standardization within studies requires matching the microbiota composition between animal groups, which becomes particularly critical when genetically engineered animals (e.g., a mouse strain with a certain knockout gene), which are bred at an animal facility, are compared with the respective wild-type controls, which are often purchased and subsequently imported from commercial animal vendors.

This task can be approached by using two different strategies, which both aim at matching the microbiomes of separate animal cohorts and therefore facilitate the use of appropriate control groups: (1) littermate or (2) co-housing method. In addition, bacterial collections and gnotobiotic approaches are developing that will enable scientists to select defined microbiomes for their models under study.

##### Littermates

The littermate approach is primarily a breeding strategy, based on obtaining all experimental animals from the same breeding stock. Depending on the required genotypes, breeding couples (obtained via a random P1 mating of the homozygous wild-type and mutant strains) which have heterozygous alleles for wild-type and mutated genes (wt/mut) will produce offsprings (=littermates) which have similar microbiome compositions while expressing different gene combinations. Due to *Mendel´s law*, 25% of the litter will express homozygous alleles for the wild-type and mutant genes (wt/wt and mut/mut) and can subsequently be used for following experiments. The major advantage of this method is that siblings will have similar microbiota from birth on, guaranteeing analogous maturation of immune functions in early life development. Furthermore, there will automatically also be the highest possible level of genetic standardization, as all animals will have a high level of genetic background homology. However, unfortunately, 50% of the offsprings cannot be used for experiments, as those animals will express heterozygous alleles (wt/mut), leading to a relatively high number of breeding excess. Furthermore, this method requires constant genotyping procedures of the colony, which can be cost and time consuming and makes (invasive) biopsy sampling necessary.

##### Co-Housing and Cross-Fostering

The co-housing of experimental groups will also facilitate the decreasing of microbiome variation. Therefore, animals have to be housed together for at least four weeks, as this period was shown to be most efficient and results in the highest levels of microbiome similarity [224]. This method is probably the easiest way to achieve microbiome adaptation; however, the time point of starting the co-housing procedure remains critical, as it works best in very young animals [225,226,227] and will eventually not work for (aggressive) male individuals. In this case, cross-fostering would be a better strategy, yet it requires the marking of animals at a very early time point or, like the littermate method, constant genotyping after weaning. It also has to be noted that co-housing will lead to the asymmetric transmission of microbiota between two mouse populations, as it was shown that the microbiota of mice obtained from the Jackson Laboratories was more abundant than the microbiota from Taconic, after finished co-housing procedure [224]. A recent study conducted by Robertson et al. compared co-housing and littermate methods and demonstrated that the use of F2 littermates was the superior strategy for microbiome standardization [228].

##### Microbiome on Demand—Gnotobiology

Gnotobiology serves as a powerful instrument to study complex microbial–host interactions during animal experiments [193]. Furthermore, the use of gnotobiotic animals associated with defined microbiota is without doubt the strictest and most definite strategy to standardize an animal’s microbiome [193,229]. For this purpose, several defined microbial consortia are available which can be used to associate breeding mice and rat colonies within isolator systems. The so-called “Schaedler flora” was first established by Russel W. Schaedler, who associated germfree animals with five bacterial cultures isolated from conventional mice [230]. The original content was later modified by R.P. Orcutt, resulting in the now commonly used “Altered Schaedler Flora” (ASF), consisting of eight microorganisms [231]. The “Simplified Human Intestinal Microbiota” (SIHUMI) consists of seven bacterial species, based on numerical importance and fermentative abilities in the human gut [232,233,234]. However, those minimal consortia cannot compensate for the complexity of natural microbial communities. In this context, *Norin* and *Midvedt* demonstrated already in 2010 that ASF colonized mice are more similar to germfree than to conventionally colonized animals [235], indicating that minimal defined consortia may result in artificial reactions and loss of disease phenotypes. The same problems may even also occur when using animals housed in strict barrier systems, as their microbiome also lacks its natural diversity and was shaped into a quite limited state, indicated by a reduced alpha diversity (richness) of consisting bacterial species [6,8,215]. Therefore, it would be optimal to have access to more complex, but defined microbial communities, to create “microbiome on demand” where scientists can choose their ideal microbiome composition, with individual inclusion or exclusion profiles. Projects such as the establishment of the “mouse intestinal bacterial collection” (miBC) will serve as an essential resource, offering access to defined bacteria and microbiological communities [236]. This includes the “Oligo Mouse Microbiota12” (OMM^12^), representing 12 members of the major bacterial phyla in the murine gut providing colonization resistance against *Salmonella typhimurium* [232,234] This consortium is currently being modified to enhance complexity or to select for certain community characteristics and might represent an ideal modular system for the future [237,238,239,240].

#### 6.2.2. Intentional Diversification of the Microbiome—Re-Wilding, Wildlings, Pet-Shop or Wild Animals

As mentioned before, artificial reactions and loss of disease phenotypes may occur in animals harboring a limited or less diverse microbiome, as is nowadays often the case in breeding colonies with “SPF” or even “SOPF” (specified opportunistic and pathogen-free) status [13]. This artificial situation may have dramatic effects on immunological functions, as *Beura* et al. proved that “SPF” laboratory mice lack certain memory T cells, which more closely resembles neonatal than adult humans [241]. The authors then made use of free-living feral and also pet-shop mice, which had a more diverse microbial experience, and could prove that these animals not only expressed a more human-adult-like T-cell profile, but were also able to induce those cell populations in the laboratory mice after co-housing them with the wild animals. Likewise, others also demonstrated that the wild mouse cellular immune system is in a more activated state, that high pathogen exposure induces the activation of myeloid cells, helping to maintain natural immune homeostasis [242,243], and that experiments on mice living in a more natural habitat or experimental modifications such as co-hosing with pet-shop mice can deliver dramatically different immune phenotypes [244,245]. Analysis of “wild mouse microbiota” revealed that its composition shows relatively high inter-individual variation, which can be principally explained by geographical location, and that it undergoes a marked seasonal variation. However, the majority of bacteria remain relatively stable upon entrance into captivity [246,247,248]. In general, it can be stated that the health status of wild rodents is more robust and that animals benefit from the high microbiota diversity, improving host fitness and disease resistance [249,250,251]. An elegant series of studies was conducted by Rosshart et al., who used the technique of embryo transfer with wild caught mice as dams to transfer desired genotypes into the desired “wildling” microbiome condition. He respected the pathogen status of wild caught animals and used only those as dams that were free of known pathogens. Therefore, the group showed that the microbiota of wild animals excellently phenocopied human immune responses and that the reproducibility of preclinical studies could be enhanced using a model composed of natural microbiota and certain pathogens [252]. An excellent review on the effect of “naturalizing mouse models”, which of course goes well beyond the microbiome as it includes biotic and abiotic factors, has recently been published by Graham [253].

Despite the fact that intentional diversification using rather extreme strategies such as re-wilding offers great opportunities for immunological research, it also has major challenges, especially with regard to the presence of potentially harmful pathogens [254,255]. As there is an ethical commitment, which is also reinforced by European animal welfare legislation, to keep the animals healthy, housing them in natural habitants has to be brought together with classical health and quality assurance, as described above. These include classical health monitoring strategies to define the pathogen status, biosafety measures to exclude zoonoses and control of disease outbreaks on the one hand and on the other hand welfare tasks such as definition of clear control intervals and of humane endpoints due infectious diseases, especially once genetically modified animals with unclear immune status are studied. Beyond that, compliance with legal requirements needs to be considered when using these techniques, e.g., daily health inspections of each animal are requested by the directive 2010/63 as well as ingress- and escape-proof enclosures. Especially the latter one becomes highly relevant for genetically engineered models. The intentional release of genetically modified organisms is strictly regulated, and such animals have to be separated from local wild populations. Therefore, studies using those diversification techniques should provide clear and comprehensive descriptions of all preventive measures to address those remaining concerns regarding animal welfare aspects and the overall genetic and biosafety concepts.

#### 6.2.3. Microbiome as a Co-Factor

Differences in the microbiome can be used to intendedly induce variation by performing multi-center studies, which have become standard in clinical studies and are evolving in preclinical research also [256], or studying models in different barriers at one research location. While both approaches are feasible with the infrastructure at hand, they come with their own costs: they still rely on phenotypes that are valid in the classical “SPF” world, they increase study complexity and animal numbers for a given study considerably, and to fully control experimental settings, routine microbiome monitoring needs to be established and become a standard. The increase in complexity is limiting especially the multi-center approach to validate major findings or models before research strategies evolve towards clinical research. Multiple barrier approaches are much easier to conduct given that different barriers with identical rodent strains are present at a facility (which require genetic standardization); however, this approach is unlikely to be performed routinely but rather for validation as described above. In addition, control of the microbiome in various barriers would present a new step in the design of a health monitoring strategy.

Since common concepts only aim at monitoring lists of defined pathogens, there is obviously a lack of knowledge about the overall microbiota composition of colonies. Including monitoring of the microbiome into routine HM procedures could help to close this gap and offer excellent opportunities to cope with the upcoming challenges. Molecular techniques such as Next Generation Sequencing could be powerful tools to achieve both pan-pathogendetection as well as monitoring microbiome compositions within complex barrier systems. Especially IVC systems could further improve from sequencing methods, as exhaust air dust filters may serve as potential targets for prospective and retrospective screening of whole microbiota communities within a colony. Since nearly all infectious agents contain specific nucleic acids, sequencing is an attractive approach for pathogen detection, as a revolutionary technology already established in humane medicine and used for the diagnosis of CNS infections, bloodstream infections, respiratory infections, orthopedic infections, gastrointestinal infections and ocular infections [195,257,258]. In veterinary medicine, metagenomic sequencing was successfully used for monitoring antimicrobial resistance in swineherds [259] using fecal samples from the pen floor. The authors of this study concluded that metagenomic read mapping outperformed cultivation-based techniques in terms of predicting expected tetracycline resistance based on antimicrobial consumption.

The major challenges of this method are the needs of the so-called host depletion methods, which have to be used to diminish the host genetic background signal in the sequencing data, so that they may not interfere with data analysis. Challenges may also occur due to very complex raw data output, which do not only require high bioinformatics expertise for successful analysis but also occupy huge storage capacities. Another drawback may be the general proneness to sample contamination, making results interpretation generally complicated [195]. Comprehensive studies will be needed to validate this method, also aiming at developing valid data bases and robust guidelines for implementing this method into routine HM concepts [260], helping responsible persons to understand both the power and limitations of this method [194].

Independent from all HM concepts and methods used for monitoring, proper reporting of results is certainly the most important task. Sharing information about an animal’s hygienic status, and, in future, their microbiome, is the essential basis for creating an environment where reproducibility is even at least possible [221]. Unfortunately, a broad number of research papers withdraws that information from the materials and methods parts, sometimes due to lack of space, sometimes due to other reasons. As most journals meanwhile became more aware of the need for a standardized reporting philosophy, guidelines were published to assist and promote the systematic transfer of information about study protocols, including the hygienic status of the animals [261,262].

## 7. Conclusions

HM of laboratory rodents evolved over the last century, as increasing competence changed the focus of the monitoring procedures. In the very beginning of domestication, primary pathogens causing more or less severe infectious diseases were the major concern of veterinarians and researchers. Extensive re-derivation processes cleared most pathogens from naturally infected colonies, not only improving the overall animal health but also making the work with animals safe with respect to zoonotic diseases. Classical strategies in the form of clinical examinations and full necropsy procedures are basic skills to keep animals healthy. Traditional microbiological, virological and parasitological diagnostic methods support those basic skills and helps with systematically defining the hygienic quality of the animals. Concrete recommendations for the HM of laboratory rodents published by the FELASA led to process harmonization and helps defining the specified pathogen status of breeding and experimental colonies, enhancing the overall quality of biomedical research. However, those recommendations should not be misinterpreted as a tool for the supervision of simple exclusion lists. Instead, they serve as a solid foundation for a risk orientated definition of pathogen screening panels, which has to be individually adjusted by including other agents, which might be relevant for the respective research projects. Here, basic diagnostic skills should be united with molecular technologies to supplement traditional monitoring programs by including the analysis of environmental sample material, such as the EAD of IVC rack systems. With increasing knowledge about the relevance of whole microbiota compositions for biomedical research data, the influence of individual microbiomes has to be respected by standardizing microbiome compositions within research studies and by including metagenomic-sequencing techniques in modern HM programs. As isolated husbandry within strict barrier systems led to the development of very clean colonies with a limited diversity of gut microbiota, researchers may have to face a loss of certain disease phenotypes in animal models. Introducing controlled microbiome variation using different barriers or microbiological diversification using re-wilded animal cohorts may improve the translational value of biomedical research studies. However, there are remaining concerns regarding genetic and biosafety measures when working with colonies housed in more natural environments. Additionally, the general ethical commitment of veterinarians and researchers to care for the animals obliges them to keep colonies healthy and free of harmful pathogens, especially when working with immune-compromised animals. A reformed monitoring approach using the metagenomic-sequencing method could combine pathogen detection with microbiome analysis, increasing the informative value of a one-sample testing method.

In the end, there is cumulative evidence that “the cleaner” is not always “the better”, and that different models require different needs for microbiota compositions. Since some immunological research will benefit from the presence of certain opportunistic agents priming special immune cell populations, known confounders have to be excluded to ensure study validity. On the contrary, immunodeficient animals have to be housed within strict opportunistic free barriers to guarantee their general health integrity. Those aspects have to be already considered at the stage of project planning and should be explicitly addressed by the respective researchers and funding institutions. Yet, it is of even greater importance to improve the overall reporting routine of the animals’ health status as well as possible impacts of individual microbiome compositions on study results. Therefore, HM is supposed to be included as an essential element in all relevant recommendations such as the PREPARE and ARRIVE guidelines [262,263]. This will increase the awareness of the scientific community that modelling matching scenarios for individual study needs is challenging and requires complex strategies. However, this is a necessary task to combine classical quality and welfare-centered measures with the demand for increasing scientific validity. Thus, laboratory animal scientists have to gain outstanding competence in modern HM concepts, continuing its (r)evolution.
